# Novel targets and therapies of metformin in dementia: old drug, new insights

**DOI:** 10.3389/fphar.2024.1415740

**Published:** 2024-05-31

**Authors:** Wenxing Cui, Chen Lv, Panling Geng, Mingdi Fu, Wenjing Zhou, Mingxiang Xiong, Tian Li

**Affiliations:** ^1^ College of Life Sciences, Northwest University, Xi’an, China; ^2^ Department of Neurosurgery, Tangdu Hospital, Fourth Military Medical University, Xi’an, China; ^3^ Hangzhou Simo Co., Ltd., Hangzhou, China; ^4^ School of Basic Medicine, Fourth Military Medical University, Xi’an, China

**Keywords:** metformin, dementia, insulin resistance, oxidative stress, neuroinflammation

## Abstract

Dementia is a devastating disorder characterized by progressive and persistent cognitive decline, imposing a heavy public health burden on the individual and society. Despite numerous efforts by researchers in the field of dementia, pharmacological treatments are limited to relieving symptoms and fail to prevent disease progression. Therefore, studies exploring novel therapeutics or repurposing classical drugs indicated for other diseases are urgently needed. Metformin, a first-line antihyperglycemic drug used to treat type 2 diabetes, has been shown to be beneficial in neurodegenerative diseases including dementia. This review discusses and evaluates the neuroprotective role of metformin in dementia, from the perspective of basic and clinical studies. Mechanistically, metformin has been shown to improve insulin resistance, reduce neuronal apoptosis, and decrease oxidative stress and neuroinflammation in the brain. Collectively, the current data presented here support the future potential of metformin as a potential therapeutic strategy for dementia. This study also inspires a new field for future translational studies and clinical research to discover novel therapeutic targets for dementia.

## 1 Introduction

Dementia is a clinical syndrome characterized by progressive cognitive deterioration accompanied by behavioral, social and emotional function disability, imposing a heavy burden on society (van der Steen et al., 2018). Approximately 50 million people had dementia worldwide in 2018; (Alzheimers Dement, 2023); is estimated to triple worldwide in 2050 and is higher in low- and middle-income countries than in high-income countries (Prince et al., 2016; Scheltens et al., 2021). Although progress in treating neuropsychiatric symptoms is being reported, the benefit is limited and temporary (Moran et al., 2019). In addition, many disease-modifying therapies for dementia are discontinued due to toxicity or futility (Cummings et al., 2020). Improving insights into the biological processes, abundant biomarkers and clinical features of dementia contribute to the discovery of new therapeutic targets or reuse of classical drugs (Scheltens et al., 2021).

The molecular pathways underlying different types of dementia primarily involve oxidative stress, mitochondrial bioenergetics, neuroinflammation, neurodegeneration, and insulin resistance (Jurcău et al., 2022; Gaikwad et al., 2024). Oxidative stress is a classic molecular mechanism (Yang et al., 2016; Yang et al., 2017; Tang et al., 2021; Zhang et al., 2023). In recent years, emerging evidence has revealed the close relationship between diabetes, cognitive dysfunction and dementia (Little et al., 2022). People with type 2 diabetes (T2D) have a 1.5- to 2-fold higher risk of dementia than those without diabetes (Gregg et al., 2000; Cukierman et al., 2005; Roriz-Filho J et al., 2009). Diabetes and prediabetes have been shown to accelerate the progression from mild cognitive impairment to dementia (Xu et al., 2010; Xing et al., 2020; Li et al., 2022). T2D and dementia share the same risk factors, such as older age, obesity, and insulin resistance (Pugazhenthi et al., 2017; Arnold et al., 2018). At the cellular level, T2D has been implicated in oxidative stress, mitochondrial dysfunction, and inflammation that are also present in individuals with dementia (Pugazhenthi et al., 2017). Considering the common risk factors and pathological mechanisms prevailing in T2D and dementia, antidiabetic drugs may exert promising protective effects on brain metabolism and dementia. Antidiabetic drugs encompass metformin, sulfonylurea, thiazolidinediones (TZD), dipeptidyl peptidase-4, GLP-1 receptor agonists, sodium-glucose cotransporter 2 inhibitors, meglitinides, and alpha-glucosidase inhibitors (Slouha et al., 2023). Metformin is the first-line drug treatment for T2D, and exerts antidiabetic effects mainly by inhibiting hepatic glucose production (Duca et al., 2015; Li et al., 2020; Li and Ma, 2020; Li et al., 2021; Li et al., 2022; Du et al., 2022). Moreover, metformin activates 5′AMP-activated protein kinase (AMPK) (Ma et al., 2016; Li et al., 2017; Rena et al., 2017; Hu et al., 2021), improves insulin resistance (Ford et al., 2015), decreases neuronal apoptosis (Li et al., 2019), and reduces oxidative stress and the inflammatory response in the brain (Obafemi et al., 2020). In recent clinical studies, the use of metformin in elderly patients with T2DM is significantly linked to a substantial decrease in the risk of dementia (Sun et al., 2024; Tang et al., 2024). In light of the important roles of metformin in peripheral and central metabolism, the present review discusses recent breakthroughs in metformin treatment of dementia.

**FIGURE 1:**
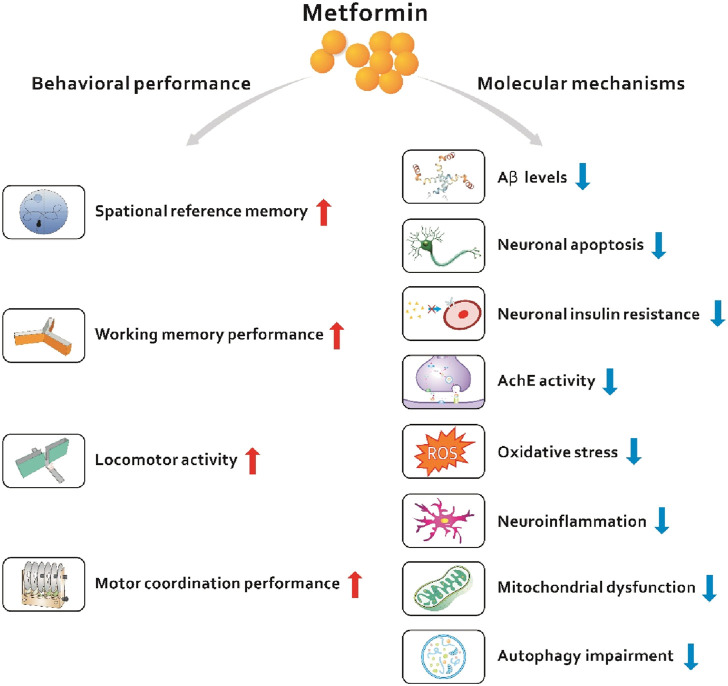
Novel target and therapies of metformin in dementia.

Based on currently published data, we speculate that metformin is a potential alternative drug candidate for the treatment of dementia. This review will first introduce the general background on dementia, mainly including Alzheimer’s disease (AD)-related dementia and T2D-related dementia, as well as the common pathways in T2D and dementia. Second, we describe the mechanisms by which metformin regulates peripheral and central metabolism in cell and animal models. Then, we summarize the clinical evidence that metformin is able to treat dementia. Finally, we propose potential research directions and provide insights into the treatment of dementia with metformin (Figure 1).

## 2 General background on dementia

### 2.1 AD and dementia

AD is the most common type of dementia in the elderly, and with the advent of the aging era, AD imposes a heavy economic and social burden worldwide (Diniz Pereira et al., 2021; Liao et al., 2021; Ning et al., 2022). According to a European memory clinic cohort, the median survival time depends on the type of dementia, and the survival time of individuals with AD-related dementia is 6.2 (6.0–6.5) years (Rhodius-Meester et al., 2019). The characteristic pathological changes of AD are neuronal fibers and axonal tangles in the brain, and the formation of large amounts of senile plaques; these changes drive neuronal dysfunction and cell death (Scheltens et al., 2021). Biomarkers for the diagnosis of AD were defined as the presence of amyloid β (Aβ) and phosphorylated tau (Jack et al., 2018). Strong evidence from a community-based cohort study suggests that advanced age and at least one APOE ε4 allele are the most powerful risk factors for AD (van der Lee et al., 2018). The risk of disease onset is doubled every 5 years after the age of 65 years, and approximately 50% of patients with AD carry the apolipoprotein E (APOE) ε4 allele (Pierce et al., 2017). Moreover, diabetes/metabolic syndrome, cardiovascular disorders and stroke are also established risk factors for AD (Tosto et al., 2016; Campos-Peña et al., 2017). In addition, stress and glucocorticoids have also recently been identified as potential factors that increase the risk of developing AD (Caruso et al., 2018). The latest perspectives noted that glucose metabolism moved to center stage in AD research (Kuehn, 2020; Park et al., 2023).

The treatment principles of AD include early diagnosis, timely treatment, and lifelong management. Although existing anti-AD drugs do not reverse the disease, they prevent cognitive decline and dementia, and patients should adhere to long-term treatment as much as possible. Currently approved drugs for the standard treatment of patients with AD include cholinesterase inhibitors and the N-methyl-D-aspartate receptor antagonist memantine (Scheltens et al., 2016). The first choice for the psychobehavioral symptoms of dementia is the nonpharmacological intervention, and psychotropic drugs can be used when necessary, but the efficacy and side effects should be assessed regularly and long-term use should be avoided. Agitation and aggression are common neuropsychiatric problems associated with AD, and brexpiprazole (an atypical antipsychotic), citalopram (a selective serotonin reuptake inhibitor) and nabilone (a cannabinoid) represent relatively safe treatment options for agitation and aggression (Liu et al., 2016). Other disease-modifying therapies for AD have also been developed. For example, aducanumab, BAN2401, and gantenerumab reduce the amyloid β plaques burden (van Dyck, 2018). Health education, psychological support and practical help can improve the quality of life of patients with AD. The future of personalized treatment for AD should include multimodal interventions, which are based on the individually customized incorporation of lifestyle changes and drugs.

### 2.2 T2D and dementia

Due to the growing elderly population, the incidence of both diabetes and neurodegenerative diseases is increasing worldwide. The relationship between diabetes and dementia is likely to be complex and multifactorial. The Rotterdam Study is the first to identify a remarkably increased risk of dementia in patients with T2D, including vascular dementia and AD (Ott et al., 1996; Ott et al., 1999). Compared with the general population, patients with T2D have a 1.5–2-fold higher risk of dementia (Gregg et al., 2000; Cukierman et al., 2005). One in every 10 to 15 cases of dementia is attributable to type 2 diabetes (Biessels et al., 2006). The mechanism of T2D-related dementia includes hyalinization of the basal membrane of cerebral arterioles due to diabetes, causing endothelial cell damage; meanwhile, the hemodynamics, vascular reactivity and autoregulation function are also affected, resulting in reduced cerebral blood flow and thereby increasing the occurrence of clinically silent cerebral infarction (Kalaria, 2002). Moreover, the metabolism of glucose, lipids and amino acids in the brains of patients with T2D may be disturbed due to factors such as poor blood glucose control, increased glycosylated hemoglobin levels, central and peripheral insulin resistance, oxidative stress and the inflammatory response, which affects the transmission of neurotransmitters and changes the homeostasis of the local microenvironment, coupled with long-term ischemia and hypoxia of the brain tissue, ultimately causing neuronal necrosis and apoptosis as well as cognitive decline (Berlanga-Acosta et al., 2020; Zhou et al., 2020; Abosharaf et al., 2024). Indeed, T2D and dementia share the same risk factors, such as older age, obesity, insulin resistance and physical inactivity. Thus, theoretically, drugs used to treat T2D could modify these risk factors and pathogenesis (Areosa Sastre et al., 2017).

### 2.3 Principal mechanisms linking T2D and AD

Although T2D appears to be primarily a peripheral organ disease and AD is a central nervous system disorder, evidence from experimental and clinical studies has indicated a close link between T2D and AD (Craft, 2009; Kuehn, 2020). A meta-analysis of diabetes and the risk of dementia included 28 prospective studies examining 89708 patients with diabetes and revealed that the relative risks of developing all types of dementia and AD were 1.73 and 1.56 in patients with diabetes, respectively (Gudala et al., 2013). First, accumulating evidence shows that glucose hypometabolism may play a key role in AD pathology (Kuehn, 2020). Insulin resistance in the brain may cause AD pathology, which has led some scientists to propose that AD may be a brain-specific “type 3 diabetes”. Insulin exerts neurotrophic effects at moderate concentrations, but excessive insulin in the brain leads to decreased Aβ clearance due to competition for the common and main clearance enzyme, insulin-degrading enzyme (IDE). Therefore, the accumulation of large amounts of Aβ in the brain due to pathological insulin levels contributes to the pathological features associated with AD (J et al., 2009). In addition, insulin may rapidly increase tau phosphorylation, which causes the accumulation of neurofibrillary tangles (NFTs) and senile plaques (Lesort and Johnson, 2000). Mechanistically, the mitogen-activated protein kinase (MAPK) pathway is activated in response to insulin receptor signaling and plays an important role in AD pathogenesis. The activation of MAPK regulates cell proliferation, is associated with Aβ plaques and NFTs and is also involved in tau phosphorylation, neuroinflammation, and synaptic plasticity (Munoz and Ammit, 2010). On the other hand, impaired glucose metabolism is considered a risk factor for AD, as evidenced by a decrease in glucose metabolism in the regions related to memory processing and learning (Mosconi et al., 2008). Chronic hyperglycemia might damage the brain through the accumulation of advanced glycation end products (AGEs) and increased oxidative stress (Vlassara and Uribarri, 2014). AGEs also accelerate the progression of AD through increases in Aβ levels, senile plaques and intracellular NFTs (Iannuzzi et al., 2014).

Second, decreases in the hippocampal volume and cortical thickness have been observed in patients with T2D, changes that are closely associated with cognitive decline (Ben Assayag et al., 2017). These harmful phenomena are potentially attributed to increased neuronal apoptosis and decreased neurogenesis. Impaired neurogenesis is associated with elevated levels of glucocorticoids and decreased expression of brain derived neurotrophic factor (BDNF), both of have been observed in patients with T2D and AD (Marosi and Mattson, 2014; Dey et al., 2017). Activation of the cyclic adenosine monophosphate (cAMP)/protein kinase (PKA) signaling pathway has been documented in AD and T2D mice and causes neuronal apoptosis (Li et al., 2018). Third, oxidative stress and neuroinflammation are two important pathological processes in T2D and AD. The brain is susceptible to an oxidative imbalance because of the attributes of a high energy demand and oxygen consumption, leading to a large number of oxidized polyunsaturated fatty acids (Mecocci et al., 2018). Oxidative stress causes peroxidation of mitochondrial membranes and enzymatic proteins, whose accumulation has been detected in the hippocampus and frontal and temporal lobes of patients with mild cognitive impairment (Zabel et al., 2018). Increased ROS generation and oxidative stress are also common in T2D (Dos Santos et al., 2018). Meanwhile, ROS-mediated oxidative stress is associated with an inflammatory phenotype (Sindhu et al., 2018). An excessive inflammatory response may lead to Aβ accumulation, Tau phosphorylation, and changes in synaptic plasticity, which lead to AD pathology (Carret-Rebillat et al., 2015; Falcicchia et al., 2020). Additionally, unresolved inflammation contributes to insulin resistant pathology, cell death, and excessive ceramide production, which subsequently aggravate inflammation (Keane et al., 2015). A meta-analysis of 170 studies revealed that peripheral inflammation is associated with AD (Shen et al., 2019). Other common mechanisms, such as blood-brain barrier (BBB) disruption (Kaminari et al., 2018), acetylcholinesterase (AChE) metabolism (Rao et al., 2007), and senescence (Palmer et al., 2015), likewise link AD and T2D closely. Pereira and his colleagues reported that 17 common biomarkers were differentially expressed in patients with AD or T2D compared with healthy controls. These biomarkers provide a strong reference for detecting patients with T2D at risk of developing AD (Diniz Pereira et al., 2021). Altogether, most of the current evidence indicates that T2D may hasten the progression of AD, and there are numerous shared mechanisms between AD and T2D.

## 3 Metformin acts as a potential protective agent against dementia

Considering the multifaceted links between T2D and dementia, researchers have good reasons to believe that antidiabetic drugs can treat dementia. Metformin, a biguanide derivative, is now widely used and a first-line therapeutic option for the treatment of T2D (Nathan et al., 2009). Metformin lowers hyperglycemia by inhibiting hepatic glucose production, improving insulin sensitivity, and increasing peripheral glucose uptake in muscle (Duca et al., 2015). In addition, metformin exerts positive effects by improving cell metabolism, decreasing neuronal apoptosis, and reducing oxidative stress and the inflammatory response in the brain. Hundreds of clinical studies have examined the protective effects of metformin on dementia, suggesting that metformin shows therapeutic potential as a treatment for dementia. Next, we will delineate the role of metformin in dementia at the basic and clinical levels.

### 3.1 Cell and animal experiments

The results of current preclinical and mechanistic studies have provided some insights into the effects of metformin on dementia. Metformin has the potential to activate the AMPK pathway, which plays a crucial role in the pathogenesis of dementia (Nikbakhtzadeh et al., 2021). There is increasing evidence suggesting that the activation of AMPK may have extensive neuroprotective effects for dementia, such as promoting autophagy, maintaining mitochondrial quality control, reducing insulin resistance, and alleviating oxidative stress (Yang et al., 2020). Some studies have provided evidence that metformin ameliorates cognitive impairment and memory loss. Allard et al. (Allard et al., 2016) found that prolonged metformin treatment prevents the high-fat diet-induced impairment in spatial reference memory in mice. Similarly, Chen et al. (2016) showed that chronic treatment of db/db mice with metformin ameliorates memory impairment, as confirmed by improved performance on behavioral tests. The generation of amyloid peptides and aggregation of abnormally folded proteins are important shared pathological characteristics of T2D and AD (Knowles et al., 2014). According to one study, metformin decreases hippocampal β-amyloid (Aβ) levels, inhibits neuronal apoptosis, and ameliorates the memory impairment in db/db mice (Chen et al., 2016). Metformin significantly decreases beta-secretase 1 (BACE1) protein expression and activity both in cell culture models and *in vivo*; this enzyme is involved in the production of Aβ(Hettich et al., 2014; Markowicz-Piasecka et al., 2017). As shown in another study by Gupta et al. (2011), metformin ameliorates neuronal insulin resistance and AD-like changes including markedly increased Aβ levels. Chakravarty and Nielsen (1986) also showed that the brains of db/db mice have multiple AD-like properties including impaired cognitive functions, increased phospho-tau and Aβ levels and decreased levels of synaptic proteins, changes that were attenuated by metformin (Li et al., 2012). In contrast to the abovementioned articles, Chen et al. (2009) found that metformin treatment of a transgenic mouse model of AD contributed to the increased expression of BACE1 in an AMPK-dependent manner, which led to an increase in Aβ production. This finding suggests a potential harmful effect on accelerating AD pathogenesis, and metformin should be used with caution in elderly patients with diabetic.

Metformin has also been shown to decrease the activity of acetylcholine esterase (AChE) and subsequently improves memory in diabetic rats. AChE is responsible for degrading acetylcholine, the main neurotransmitter involved in learning and memory processes (Bhutada et al., 2011). A recent study found that metformin might preserve hippocampal synaptic plasticity, inhibit AChE activity, and normalize acetylcholine clearance (Pilipenko et al., 2020). These data indicate a promising protective effect of metformin on severe cognitive decline. Many studies have revealed a pivotal role for oxidative stress in the pathological process of dementia, which subsequently increases the levels of its markers, such as oxidized lipids and proteins (Butterfield et al., 2006). The oxidation of proteins contributes to impaired cerebral glucose metabolism in AD, which in turn results in neuronal degeneration and cognitive deficits (Chen and Zhong, 2013). In addition, oxidative stress promotes Tau hyperphosphorylation (Sultana et al., 2006). Obafemi et al. (2020) found that metformin significantly reduces the levels of malondialdehyde and increases the activities of SOD, GPx and catalase. Moreover, the levels of ER stress markers are attenuated in the hippocampus. These results indicate the inhibitory effect of metformin on diabetes-induced oxidative stress. In addition to oxidative stress, the inflammatory response also plays a major role in the development and progression of T2DM and AD (Mushtaq et al., 2015). Lu et al. (2020) showed that metformin decreases neuroinflammation (IL-1 and IL-6) and oxidative stress (MDA and SOD) in APP/PS1 transgenic mice, thereby improving learning and memory abilities. Mitochondrial dysfunction has been proposed as an important process in the etiology of dementia and is closely associated with oxidative stress and the inflammatory response (Feng et al., 2024). Ruegsegger et al. (2019) observed high-fat diet-induced brain insulin resistance in mice with decreased oxidative enzyme activities, resulting in the accumulation of oxidatively damaged mitochondrial proteins and increased mitochondrial fission, which were counteracted by metformin treatment. These results suggest that metformin might restore brain mitochondrial function in the pathological insulin-resistant state.

Findings from other mechanistic studies showed that metformin treatment is closely associated with neuronal survival. Li et al. (2019) found that metformin inhibits apoptosis and decreases intracellular Ca and ROS signaling by reducing the neurotoxicity of excitatory amino acids in Aβ -treated SH-SY5Y cells. Moreover, Chen et al. (2016) reported that metformin alleviates Aβ-induced apoptosis in cultured hippocampal neurons in a JNK-dependent manner. In an *in vivo* study, metformin decreased neuronal loss in the hippocampus, enhanced neurogenesis, and attenuated spatial memory deficits in APP/PS1 mice (Ou et al., 2018). Another similar study also showed that metformin enhances neuronal survival and improves spatial memory in a mouse model of neurodegeneration (Ahmed et al., 2017). Metformin also has shown the promise in slowing age-related cognitive impairment by alleviating microglial activation and enhancing autophagy in the hippocampus. However, metformin treatment does not change neurogenesis or neosynaptogenesis in the hippocampus, suggesting that metformin does not improve cognitive function (Kodali et al., 2021). BBB permeability was seen in AD patients in clinical studies using dynamic contrast-enhanced magnetic resonance imaging (MRI) (Starr et al., 2009). Metformin has been shown to protect endothelial cell tight junction, prevent damage to the BBB through the activation of AMPK and inhibition of NF-κB (Zhao et al., 2016). In another study, Takata et al. (Ismail Hassan et al., 2020) also found that metformin upregulates the expression of ZO-1, occludin, and claudin-5 in brain microvascular endothelial cells via AMPK activation.

### 3.2 Human studies

The results from human studies have provided evidence that metformin prevents cognitive decline or dementia (Barbera et al., 2024; Doran et al., 2024). A cohort study utilizing UK primary healthcare records, involving 211,396 individuals, revealed that the use of metformin was linked to a reduced risk of dementia (adjusted HR = 0.86) and mild cognitive impairment (adjusted HR = 0.92) (Doran et al., 2024). In a cohort study of 12,220 metformin users, including 12,220 early terminators and 29,126 routine users, discontinuation of metformin treatment was found to be associated with an increased incidence of dementia. This association was largely independent of changes in HbA1c levels and insulin usage (Zimmerman et al., 2023). A longitudinal observational study involving 1393 participants found that the use of metformin was significantly associated with a reduced risk of dementia in individuals with type 2 diabetes, particularly those without neuropsychiatric disorders and non-steroidal anti-inflammatory drug use (Tang et al., 2024). Another large epidemiological clinical study from the Taiwan Health Insurance database, patients with T2D who took the antidiabetic drug metformin exhibited a remarkably decreased the risk of dementia compared with patients treated without medication after adjustment for cerebrovascular disease (Hsu et al., 2011). In the population-based Singapore Longitudinal Aging Study, older people with diabetes receiving metformin (*n* = 204) had a lower risk of cognitive decline (OR 0.49, 95% CI 0.25–0.60) than those not receiving metformin (*n* = 161). At the same time, individuals receiving metformin for more than 6 years experienced a lower level of cognitive decline than those receiving metformin for less than 6 years, suggesting that long-term metformin treatment may decrease the risk of cognitive impairment (Ng et al., 2014). A large retrospective cohort study of US veterans over 65 years of age with T2D found that metformin treatment was associated with a lower subsequent dementia risk than sulfonylurea treatment in veterans <75 years of age (HR 0.67, 95% CI 0.61–0.73) (Orkaby et al., 2017). Similarly, 8276 patients with diabetes presenting with dementia and 8276 matched patients with diabetes but without dementia were included in a large population study from German. Metformin prescribed as a monotherapy (OR  0.71, 95% CI 0.66–0.76) or as dual therapy with sulfonylureas (OR  0.90, 95% CI 0.89–0.92) was associated with a decrease in the risk of subsequent dementia (Bohlken et al., 2018). More recently, a large prospective observational study, the Sydney Memory and Ageing Study, found that older people with diabetes receiving metformin experienced slower cognitive decline and lower dementia risk. Incident dementia was significantly higher in the nonmetformin group than in the group receiving metformin (OR 5.29, 95% CI 1.17–23.88) (Samaras et al., 2020).

Pilot data from a randomized placebo-controlled crossover study showed that metformin penetrates the blood-brain barrier and improves learning, memory and attentional abilities in nondiabetic patients with mild cognitive impairment or mild dementia due to AD, although it did not exert a measurable effect on CSF AD biomarkers (Koenig et al., 2017). However, this exploratory study has some limitations including the limited sample size (20 subjects) and relatively short length of the trial (16 weeks). These positive findings are promising, especially in subjects with AD but without T2D, and warrant further exploration with larger sample sizes and longer time spans.

A comparison the efficacy (pro-cognitive effects) different antidiabetic agents for dementia and mild cognitive impairment is interesting. A network meta-analysis including nineteen eligible studies (*n* = 4855) was conducted to evaluate the effects of 6 different antidiabetic drugs (intranasal insulin, pioglitazone, rosiglitazone, metformin, sitagliptin and liraglutide) on dementia (Cao et al., 2018). Cao and others showed that the greatest pro-cognitive efficacy for 15–30 mg of pioglitazone compared to the placebo. However, the included studies have a high risk of bias, and the current analysis did not investigate moderating factors such as age, sex, and the ApoE ε4 allele, which weakens the reliability of the conclusion to some extent. A recent nationwide real-world longitudinal study (*n* = 701193) found that compared with metformin + sulfonylurea, metformin + dipeptidyl peptidase-4 inhibitor and metformin + thiazolidinediones were associated with a significantly lower risk of AD (HR = 0.922 and 0.812), suggesting that adding thiazolidinediones or dipeptidyl peptidase-4 inhibitor instead of sulfonylurea as second-line antidiabetic treatment contributed to delaying or preventing dementia (Kim et al., 2021).

In a cross-sectional study of 350 late middle-aged adults without dementia, the use of diabetes medication (with metformin being the most commonly used) was associated with reduced brain Aβ burden as determined by Positron Emission Tomography imaging (Luchsinger et al., 2020). In an analysis of investigating relationships among T2D treatment and AD biomarkers, McIntosh and others found that T2D treatment was related to lower CSF levels of p-tau, t-tau, and p-tau/Aβ1-42 when compared to untreated persons with T2D (McIntosh and Nation, 2019). Due to the limited sample size, the aforementioned studies did not individually investigate a specific therapeutic drug; however, it is worth noting that metformin is the most frequently utilized diabetes medication in these studies. Subsequent research endeavors should focus on examining the impact of metformin treatment on dementia-related markers in order to gain further insights into its effects.

Notably, however, other clinical evidence has shown that metformin treatment might increase the risk of dementia. For example, in the well-established UK General Practice Research Database (GPRD), long-term use of metformin was associated with a higher risk of developing AD-related dementia compared with no metformin use (OR 1.71, 95% CI 1.12–2.60) (Imfeld et al., 2012). Nevertheless, long-term use of sulfonylureas, thiazolidinediones, or insulin was not associated with an increased risk of developing AD. Another cross-sectional observational study showed that individuals with self-reported T2D who were taking metformin had worse cognitive performance than those who were not taking the drug (OR 2.23, 95% CI 1.05–4.75) (Moore et al., 2013). One explanation for this finding may be the lack of vitamin B12 due to the use of metformin. However, the small size of the sample, insufficient information regarding the duration of metformin use and the duration and severity of diabetes raised doubts about the validity of the findings. Thus, prospective and controlled trials are needed to explore the association between diabetes, dementia, and the effect of metformin therapy, as well the possible improvements in cognitive performance mediated by vitamin B12 supplementation. More recently, findings pooled from 5 population-based cohorts showed no significant association between metformin use and cognitive function, dementia prevalence, or brain structure (Weinstein et al., 2019). Overall, currently published data suggest a protective effect of metformin treatment on the brain, but further clinical trials are needed to support this conclusion.

## 4 Potential directions

Based on the current studies, we speculate that metformin exerts multidirectional effects on dementia (Feng et al., 2016; Zhang et al., 2017; Xin et al., 2019; Li et al., 2021; Zhang et al., 2021). However, many mixed conclusions have been reported, showing that metformin does not protect against dementia or even enhances the development of dementia. Well-designed, multicenter randomized and controlled clinical studies must be conducted to explore the effects of metformin on dementia. In addition, a high-quality Cochrane systematic review and meta-analysis is needed to provide a high level of evidence. As mentioned above, vitamin B12 deficiency may be an important reason why metformin promotes the development of dementia. Therefore, future clinical trials are needed to observe the effect of metformin on dementia in the presence of vitamin B12 supplementation. Next, metformin quickly crosses the blood-brain barrier and reaches various regions of the brain. In view of this biological property, metformin is a relatively good and appropriate drug candidate for neurodegenerative diseases such as dementia. However, little is known about what concentration of metformin reaches various regions of the brain and what is the most appropriate concentration needed. The safety and efficacy of the use of metformin in patients with different types of dementia must be developed. On the other hand, the biological activity of metformin is reduced after oral administration, and its structure should be modified to improve the absorption rate. It is essential to conduct further research on the impact of metformin on brain metabolism, cell signaling, inflammation, and autophagy, particularly in relation to its potential impact on insulin signaling regulation in brain. Considering the use of metformin in combination with other drugs or treatments, we should determine whether the combination of drugs can improve the management of dementia. Moreover, although the use of metformin alone does not induce hypoglycemia under normal circumstances, the potential side effect of hypoglycemia cannot be overlooked when considering hypoglycemic drugs for conditions such as dementia. In elderly individuals, falls resulting from hypoglycemia can have severe consequences, and patients should be advised to use these medications only when there is strong evidence of benefit for dementia. Future studies in the design of such drugs should consider the mechanism of such drugs, such as regulating the insulin pathway, having minimal effect on blood glucose (or stabilizing blood glucose within a reasonable range) (Huang et al., 2023).

Current research on dementia focuses mainly on elderly individuals because dementia mainly occurs in this population. Research data on the younger patients with dementia are lacking. Evidence for the efficacy of metformin in the treatment of dementia in younger people is also lacking. The mechanism of dementia is age-related; for example, intraneuronal amyloid levels increase 30–50-fold from young to old ages (Brewer et al., 2020). Extensive studies have been performed to ensure that people with dementia receive an accurate diagnosis and treatment of their condition. Future research studies should also focus on the prevalence of dementia in younger age groups and whether metformin exerts a protective effect on younger people with dementia.

Finally, the potential influence of metformin on aging mechanisms may be the basis for its overall protective effects against age-related neurodegenerative diseases. Human observational data supports the role of metformin in preventing age-related decline, and molecular analyses of septuagenarians treated with metformin indicate that it modulates multiple biological pathways in aging (Kulkarni et al., 2018). The properties of metformin will garner significant attention from the research and industry for the development of indications for metformin as an anti-aging therapeutic in humans. Aging is a complex process, and individuals within the same population may exhibit varying responses to metformin. Therefore, it is necessary to conduct large-scale, multicenter, randomized, placebo-controlled trials in order to further investigate the anti-aging effects of metformin.

## 5 Conclusion

Metformin, the most frequently used first-line antidiabetic drug, exerts a strong protective effect on cognitive impairment. These beneficial properties of metformin might stem from its molecular mechanism, including improved insulin resistance, decreased neuronal apoptosis, and reduced oxidative stress and inflammatory responses in the brain. Here, we proposed that metformin is a potential drug candidate for dementia. Based on the current studies, we 1) introduced the general background of dementia, including AD-related dementia and T2D-related dementia; 2) summarized the common principal mechanisms linking AD and T2D; 3) described the effects of metformin on dementia in cells, animals, and humans; and 4) provided potential research directions. Overall, metformin, with its rich properties that modulate multiple pathways, is a possible and attractive candidate for the prevention of neurodegenerative diseases such as dementia; however, further large-scale clinical randomized controlled studies are warranted to ensure its success.
